# Letter to the Editor. Re: “[Dataset of breast ultrasound images by W. Al-Dhabyani, M. Gomaa, H. Khaled & A. Fahmy, Data in Brief, 2020, 28, 104863]”^☆^

**DOI:** 10.1016/j.dib.2023.109247

**Published:** 2023-05-19

**Authors:** Anna Pawłowska, Piotr Karwat, Norbert Żołek

**Affiliations:** Institute of Fundamental Technological Research Polish Academy of Sciences, Pawinskiego 5B, 02-106 Warsaw, Poland

## 1 Overview

In an interesting article previously published in Data in Brief [1], the authors presented a dataset of breast ultrasound images containing lesions. As of April 22, 2023, this study has garnered significant attention from researchers, as evident by its 298 citations in Scopus data. This is unsurprising considering that the study presents one of the few publicly available datasets on breast ultrasound images, as well as binary masks highlighting the lesions. When implementing various aspects of explainable AI, we verify the correctness of the input data at every stage, especially when using various data sources. In an attempt to use this dataset for research, we did some exploration and identified some inconsistencies that could have a significant impact on the results of the studies utilizing them. As the role of tumor detection is indisputable we feel obliged to point attention to some aspects that need to be kept in mind while using this database in order to receive reliable and good quality results.

## 2 Details

A test comparing all pairs of images using the *FindGeometricTransform* function of Mathematica software [2] was used for preliminary similarity analysis of the images included in the dataset. The source code is included in Appendix A. All images were then verified visually and grouped according to various characteristics (e.g. occurrence of foreign bodies like biopsy needle, annotations in the region of interest, imaged other areas like axilla). In the following analysis numbering of cases was modified to have a single continuous set of images. Numbers 1-437 belong to the benign subset, 438-647 correspond to malignant cases and 648-780 to normal breast tissue images.

Numerical superimposition of similar images by applying affine transformations also allowed the comparison of binary masks of lesions. Examples are shown in Table 1. Green and red (red color sometimes shifts into orange due to transparency applied) areas indicate differences in masks for the same tumor image, yellow color - a common part of the masks. All identified duplicated cases of the breast lesion images (235 items) are listed in Appendix B (Table B1). Despite the slight differences in images 441-446 (not due to geometric transformations, but to the fact that they look like a time series of images recorded during the same measurement), they belong to a series of similar images of the same tumor. Thus they were also classified here as duplicates. We have added supplementary material to this paper showing all detected duplicated images [3].

**Table 1:** Examples of duplicates with aligned masks. Green and red/orange areas indicate differences in masks for the same tumor image, yellow color - a common part of the masks.

**Fig. 1:**
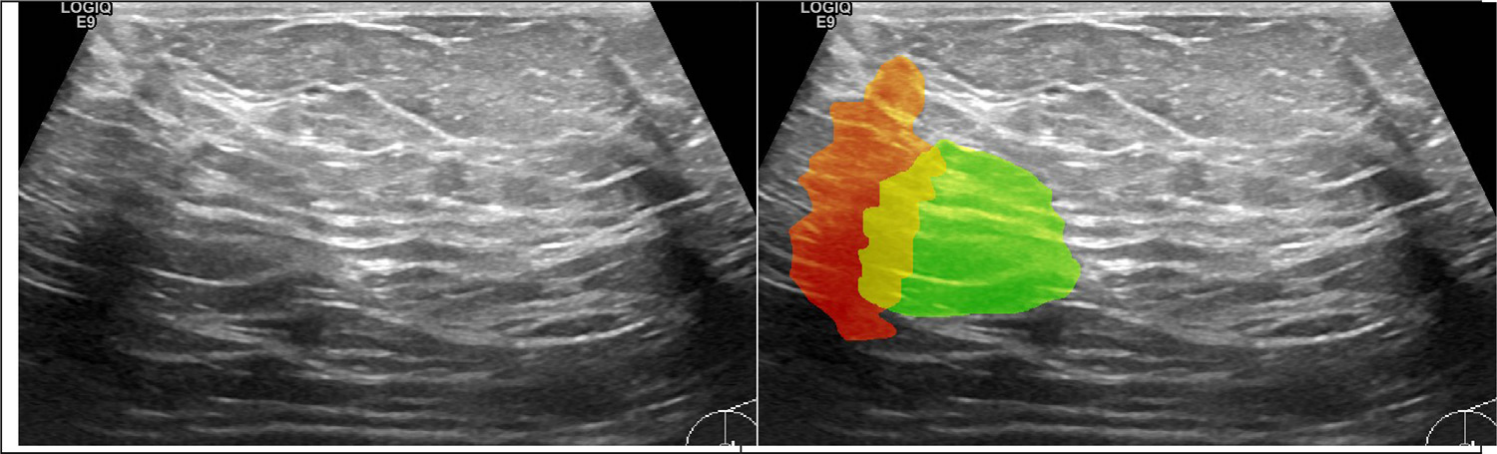
Discrepancies in reference masks in duplicated images.

After the superimposition of slightly different images and their masks, remarkable discrepancies can be seen in ground truth references describing the location and shape of the lesion (Fig. 1).

**Fig. 2:**
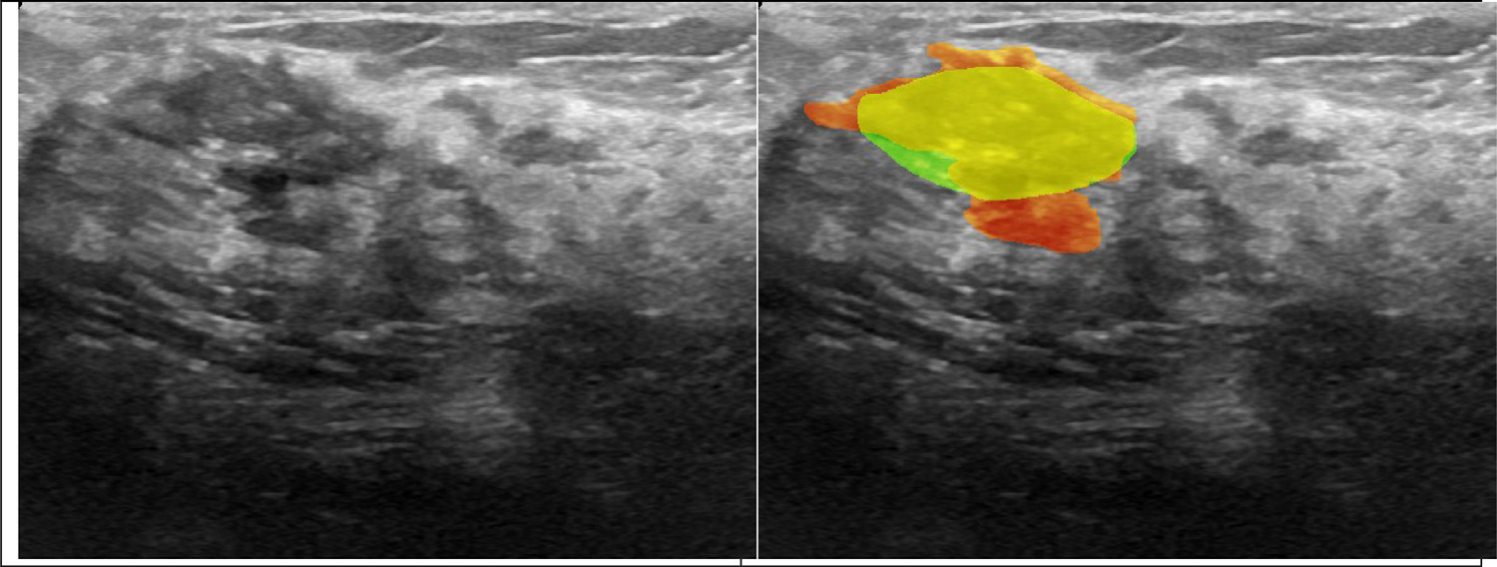
Example of duplicates in benign (42 - mask green+yellow) and malignant (488 - mask red/orange+yellow) subsets.

Even greater uncertainty occurs when the same lesion image appears in both benign and malignant collections (example shown in Fig. 2). All eight such cases are listed in table in Appendix B (Table B2). Additionally, there are two duplicates between benign and normal groups.

**Fig. 3:**
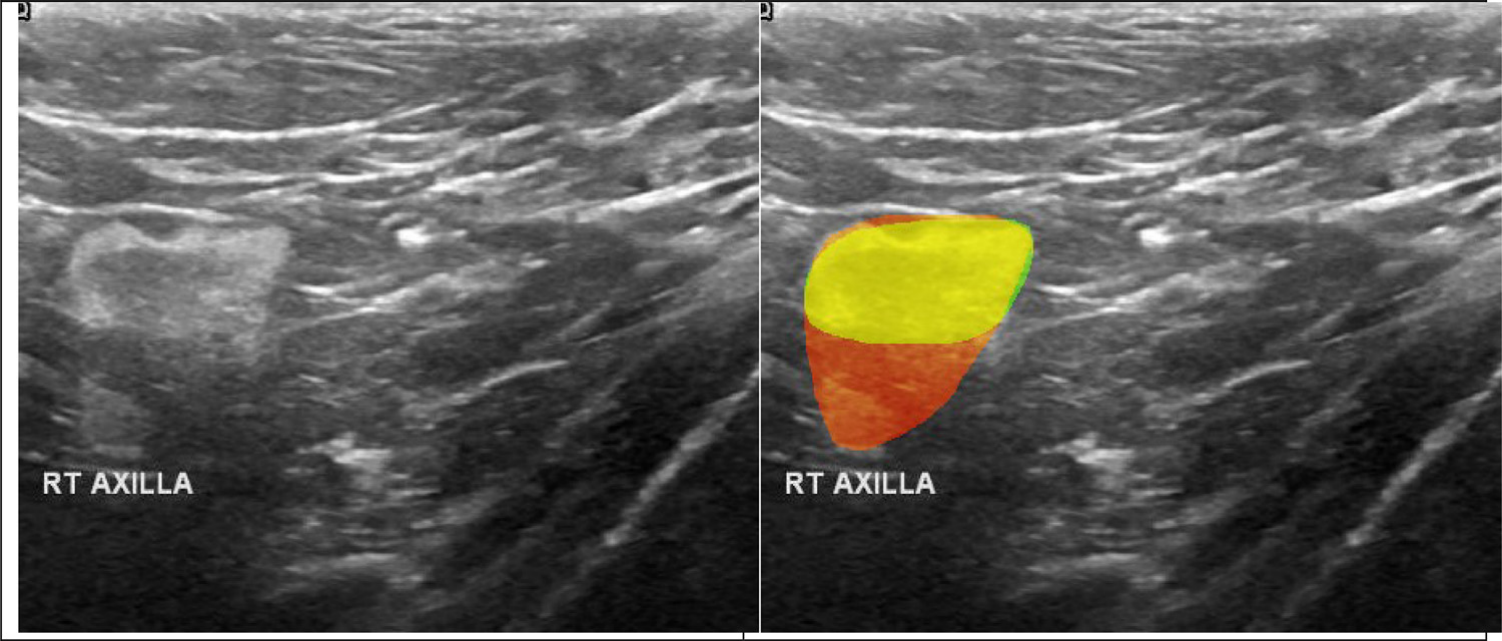
Example of duplicated images (210, 298) from right axilla with aligned masks.

**Fig. 4:**
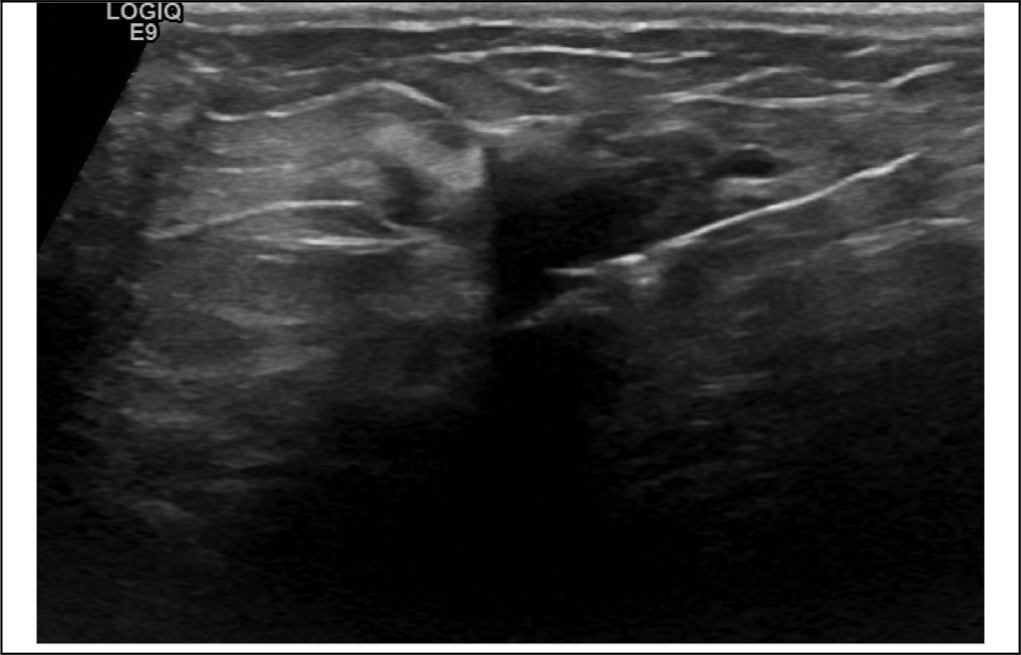
Example image with visible needle during biopsy (506).

Moreover, a significant part of the collection contains images not from the breast itself but from the axilla. It is not mentioned by the authors, although some of these images are annotated as shown in Fig. 3. We have identified 70 such cases (benign - 28, malignant - 15, normal - 27) and they are listed in Appendix B (Table B3). The images (at least 7) with a biopsy needle are also present in the dataset (Fig. 4 and Table B4 in Appendix B).

Many of the images have text or graphical annotations (dimensioning of detected lesions), which makes them difficult to use without additional pre-processing. Various types of annotations were found: text, overlay (text overlaying the tumor), measurement, doppler, pictogram and the number of their occurrences is shown in Table 2. These annotations are important to indicate since they often overlap the region of the lesion introducing significant disruptions in the image analysis process (especially in machine learning when measurements point tumor directly).

**Table 2:** Occurences of various types of annotations present in images.

	benign	malignant	normal	TOTAL
text	101	20	5	126
overlay	13	3	0	16
measurement	96	31	0	127
doppler	10	1	0	11
pictogram	6	6	3	15

The detailed classification of images is shown in Table 3.

**Table 3:** Summary of original database analysis.

	original number of cases	number of multiplicated images	number of axilla images	number of images with biopsy needle	number of multiple-classified images	TOTAL
benign	437	103	28	0	10	296 (205 without annotations)
malignant	210	22	15	5	7	161 (127 without annotations)
normal	133	30	27	2	2	72 (67 without annotations)
TOTAL	780					529 (399 without annotations)

In summary, 155 images (similar images are included - see supplementary material and images marked with purple color [3]) are copies of other images in the dataset accounting for 19% of the total collection. The 70 images (more than 8% of the entire collection) show other structures than the breast. The type of the lesion (normal, benign, malignant) of at least 19 images (more than 2%) is questionable. A detailed description of all cases is included in Appendix B. Removal of the listed inaccuracies reduces the size of the collection to at most 529 images. It should also be noted that a large number of images contain annotations (dimensions, descriptions) in the area of interest, after removing them, only 399 cases will remain.

For further research a csv file has been attached to this publication to facilitate the selection of the images that meet the relevant criteria established in this study [3]. The file consists of four columns. The first column (“ID”) contains the image number according to the numbering used in this article, in the second column (“Filename”), the filenames from the original dataset are included. The "&" sign separates multiplied occurrences of the same image. In columns 3 (“Objection”) and 4 (“Annotation”), the descriptive characteristics of images or additional annotations in images are added.

## 3 Suggestions

The original dataset requires removing duplicates as they may lead machine learning models to overlearn some patterns and result in false predictions. Moreover, randomly splitting this dataset for model evaluation into training and testing subsets – which is most common approach, a scenario with the same image in the testing and training subsets would bias the outcomes. This could potentially inflate the model performance and result in higher reported classification effects due to information leakage [4]. It is difficult to precisely state the extent to which the highlighted issues affected the results achieved in the citing publications. Authors who used this dataset will now be able to thoughtfully and thoroughly revise their results and the developed methods. To ensure the reliability of the study as well as data integrity, it is crucial to validate all results obtained from analyzing this dataset by taking into account the concerns highlighted in this letter.

## CRediT authorship contribution statement

**Anna Pawłowska:** Methodology, Investigation, Formal analysis, Writing – original draft. **Piotr Karwat:** Investigation. **Norbert Żołek:** Methodology, Software, Conceptualization, Writing – original draft, Writing – review & editing, Supervision, Project administration.

## Declaration of Competing Interest

The authors declare that they have no known competing financial interests or personal relationships that could have appeared to influence the work reported in this paper.

## Figures and Tables

**Table B1:** List of duplicates (for corresponding images see suplementary material [3]).

{{1, 318}, {2, 319}, {3, 320}, {4, 321}, {5, 322}, {6, 323}, {7, 324}, {8, 325}, {9, 326}, {10, 327}, {11, 328}, {12, 329}, {13, 330}, {14, 331}, {15, 332}, {16, 151}, {17, 425}, {18, 152}, {19, 150}, {21, 154}, {25, 153}, {30, 128}, {33, 130}, {35, 177}, {37, 127}, {38, 132}, {42, 131}, {42, 488}, {44, 129}, {50, 136}, {51, 133}, {52, 134}, {58, 135}, {60, 138}, {61, 94}, {62, 140}, {64, 141}, {65, 99}, {65, 139}, {65, 157}, {81, 197}, {85, 164}, {85, 489}, {86, 163}, {94, 108}, {96, 155}, {99, 139}, {99, 157}, {105, 156}, {108, 114}, {110, 158}, {114, 116}, {116, 119}, {119, 122}, {125, 126}, {131, 488}, {139, 157}, {164, 489}, {199, 248}, {200, 274}, {200, 290}, {201, 302}, {202, 301}, {203, 273}, {203, 289}, {204, 249}, {204, 282}, {205, 262}, {206, 303}, {207, 256}, {208, 291}, {209, 275}, {209, 297}, {210, 298}, {211, 254}, {213, 253}, {214, 260}, {214, 281}, {215, 270}, {216, 265}, {217, 268}, {218, 296}, {219, 272}, {220, 271}, {221, 246}, {222, 267}, {223, 257}, {224, 304}, {225, 292}, {225, 293}, {226, 263}, {226, 278}, {227, 250}, {228, 306}, {229, 259}, {229, 280}, {230, 252}, {231, 295}, {232, 276}, {232, 287}, {233, 299}, {234, 261}, {235, 294}, {236, 247}, {237, 264}, {238, 300}, {239, 258}, {240, 255}, {240, 277}, {241, 266}, {241, 279}, {242, 288}, {244, 251}, {245, 305}, {249, 282}, {255, 277}, {259, 280}, {260, 281}, {263, 278}, {266, 279}, {269, 648}, {273, 289}, {274, 290}, {275, 297}, {276, 287}, {284, 285}, {292, 293}, {307, 419}, {308, 424}, {309, 422}, {310, 423}, {312, 421}, {316, 426}, {333, 514}, {333, 515}, {395, 411}, {396, 413}, {399, 530}, {400, 412}, {404, 415}, {406, 531}, {433,582}, {437, 681}, {441, 442}, {441, 444}, {441, 445}, {441, 446}, {442, 444}, {442, 445}, {442, 446}, {443, 444}, {443, 445}, {443, 446}, {444, 445}, {444, 446}, {445, 446}, {447, 549}, {448, 550}, {449, 547}, {450, 546}, {451, 551}, {451, 560}, {452, 548}, {454, 525}, {455, 553}, {464, 465}, {471, 529}, {476, 479}, {502, 503}, {514, 515}, {517, 518}, {522, 523}, {532, 533}, {532, 534}, {533, 534}, {535, 536}, {543, 544}, {551, 560}, {555, 556}, {565, 566}, {652, 660}, {666, 778}, {667, 779}, {668, 710}, {668, 711}, {669, 772}, {670, 728}, {671, 775}, {672, 769}, {673, 777}, {674, 771}, {675, 773}, {676, 770}, {677, 766}, {678, 768}, {679, 774}, {680, 767}, {682, 776}, {685, 690}, {685, 697}, {685, 700}, {686, 695}, {686, 703}, {687, 693}, {687, 706}, {688, 691}, {688, 694}, {688, 708}, {689, 692}, {689, 696}, {689, 709}, {690, 697}, {690, 700}, {691, 694}, {691, 708}, {692, 696}, {692, 709}, {693, 706}, {694, 708}, {695, 703}, {696, 709}, {697, 700}, {698, 699}, {698, 701}, {699, 701}, {702, 704}, {702, 714}, {702, 715}, {704, 714}, {704, 715}, {705, 707}, {705, 716}, {707, 716}, {710, 711}, {714, 715}, {744, 745}, {751, 754}}

**Table B2:** Images present in two different subsets.

benign - malignant	benign - normal
{{42, 488}, {85, 489}, {131, 488}, {164, 489}, {333, 514}, {333, 515}, {399, 530}, {406, 531}, {433, 582}}	{{269, 648},{437, 681}}

**Table B3:** list of image numbers suspected as originated outside the breast itself (images from axilla).

benign (28)	malignant (15)	normal (27)
106, 166, 199, 205, 207, 210, 217, 218, 223, 225, 228, 233, 235, 236, 243, 247, 248, 256, 257, 262, 268, 292, 293, 294, 296, 298, 299, 306	448, 450, 545, 546, 547, 550, 586, 637, 585, 449, 617, 464, 465, 498, 604	649, 686, 689, 692, 695, 696, 703, 705, 707, 709, 716, 722, 730, 732, 737, 738, 739, 740, 741, 744, 745, 747, 748, 751, 754, 757, 765

**Table B4:** List of images with visible biopsy needle.

506, 507, 610, 627, 611, 682, 776

## Appendix A

Code in Mathematica language for preliminary detection of possible duplicates:

 (* data loading and rescaling to speedup process *)

 X = ImageResize[Import[*dataDirectory* <> "/" <> #], {160, 160}] & /@ SortBy[FileNames["*).png", {"benign", "malignant", "normal"}], ToExpression@StringDrop[FileNameTake[#], -4] &];

duplicates = {};

Monitor[

   (* compare all pairs of images *)

   For[i = 1, i < 780 (* number of images *), i++,

     For[j = i + 1, j <= 780, j++,

       (* try to find geometrical transform between images and calculate error *)

       s = Quiet@Check[ {error, transform} = FindGeometricTransform[X[[i]], X[[j]]], Null];

       If [s === Null || error < 10^-10 (* basic threshold *)

         **duplicates** = AppendTo[**duplicates**, {i, j, error}]]

     ]

   ],

{i, j}]

(* print the similar pairs of images *)

Map[{#, Part[X, #]} &, duplicates[[1;2]]]
